# The Role of Folate Transport in Antifolate Drug Action in Trypanosoma brucei*

**DOI:** 10.1074/jbc.M116.750422

**Published:** 2016-10-04

**Authors:** Simon Dewar, Natasha Sienkiewicz, Han B. Ong, Richard J. Wall, David Horn, Alan H. Fairlamb

**Affiliations:** From the Division of Biological Chemistry and Drug Discovery, Wellcome Trust Building, College of Life Sciences, University of Dundee, Dundee DD1 5EH, Scotland, United Kingdom

**Keywords:** drug resistance, folate, RNA interference (RNAi), transport, Trypanosoma brucei, RIT-seq, antifolate, human African trypanosomiasis, methotrexate, uptake

## Abstract

The aim of this study was to identify and characterize mechanisms of resistance to antifolate drugs in African trypanosomes. Genome-wide RNAi library screens were undertaken in bloodstream form *Trypanosoma brucei* exposed to the antifolates methotrexate and raltitrexed. In conjunction with drug susceptibility and folate transport studies, RNAi knockdown was used to validate the functions of the putative folate transporters. The transport kinetics of folate and methotrexate were further characterized in whole cells. RNA interference target sequencing experiments identified a tandem array of genes encoding a folate transporter family, TbFT1–3, as major contributors to antifolate drug uptake. RNAi knockdown of TbFT1–3 substantially reduced folate transport into trypanosomes and reduced the parasite's susceptibly to the classical antifolates methotrexate and raltitrexed. In contrast, knockdown of TbFT1–3 increased susceptibly to the non-classical antifolates pyrimethamine and nolatrexed. Both folate and methotrexate transport were inhibited by classical antifolates but not by non-classical antifolates or biopterin. Thus, TbFT1–3 mediates the uptake of folate and classical antifolates in trypanosomes, and TbFT1–3 loss-of-function is a mechanism of antifolate drug resistance.

## Introduction

The neglected tropical disease human African trypanosomiasis (HAT),^3^ also known as African sleeping sickness, threatens ∼70 million people in 24 sub-Saharan African countries (1). HAT is primarily transmitted by the tsetse fly and is caused by two subspecies of the unicellular protozoan parasite *Trypanosoma brucei*: *T. brucei gambiense*, an anthroponotic disease mainly affecting humans with a minor animal reservoir; and *T. brucei rhodesiense*, a zoonotic disease affecting mainly animals with humans inadvertently infected. Both forms of HAT clinically evolve in two stages, an early hemolymphatic stage and a second meningoencephalitic stage, with a fatality rate close to 100% if left untreated (2, 3). *T. brucei rhodesiense* HAT is an acute disease that rapidly progresses to death within 6 months (4), whereas *T. brucei gambiense* HAT has a more chronic course with an average duration of 3 years (5). Although new cases of HAT have fallen to below 7,000 in 2011, the disease carries a major risk of resurgence with epidemiological population shifts, climate change, and civil unrest (1, 6). The disease is a key factor in maintaining the poverty cycle in neglected communities, and it is also a stigmatizing disease causing neuropsychological impairment and abandonment for its suffers (7, 8). In 2012, the World Health Organization targeted elimination of *T. brucei gambiense* HAT (which accounts for 98% of HAT cases) by 2020 (9).

Current drugs used for the treatment of HAT are far from ideal with many shortcomings, such as high cost, severe toxicity, and the emergence of resistance (10). Nifurtimox/eflornithine combination therapy is the newest treatment to be used clinically, but the ease of resistance developing in the field is a concern (11, 12). Development of new drugs could enable the elimination of *T. brucei gambiense* HAT. Essential metabolic pathways of the parasite are being exploited to identify potential drug targets, and folate metabolism is one such pathway. *T. brucei*, like all trypanosomatids, is auxotrophic for folates (13, 14), and some of the enzymes responsible for intracellular folate metabolism have been investigated as potential drug targets. For example, the bifunctional enzyme dihydrofolate reductase-thymidylate synthase (DHFR-TS) and pteridine reductase 1 (PTR1) have been found to be essential for parasite survival, and potent inhibitors of TbDHFR-TS and TbPTR1 have good antitrypanosomal activity (13–17).

In addition, the classical antifolates methotrexate (MTX) and raltitrexed (RTX) were found to have nanomolar potency against *T. brucei* when tested in culture media with a folate concentration similar to that in human serum (13, 14). Utilization of folate-depleted medium for screening antifolates is necessary as the standard media for *T. brucei* cell culture, HMI9-T medium, contains folate at 440 times (range 140–2,000) the plasma concentration in humans (18). The impact of this high non-physiological folate concentration could reduce the antitrypanosomal potency of antifolate inhibitors through competition for drug uptake, interference with cellular retention by competition for polyglutamylation by folylpolyglutamate synthase (FPGS), or through competition for the active site of target enzymes (*e.g.* DHFR-TS).

We used a genome-wide RNA interference screening approach to identify potential antifolate resistance mechanisms, an approach that has been used successfully for other antitrypanosomal compounds (19, 20). Here, MTX (a DHFR and PTR1 inhibitor) and RTX (a TS inhibitor) were chosen as model antifolates as these drugs display potent activity against *T. brucei* (13, 14). We report the role of the folate transporter gene family (FT1–3), identified in our RIT-seq screen, in mediating folate uptake. Subsequently, we characterize the kinetics of folate and MTX transport and demonstrate substrate competition between folate and MTX. Our experiments also distinguish between classical and non-classical antifolate entry into trypanosomes and suggest a role of *p*-aminobenzoylglutamate (pABA-Glu) in facilitating the uptake of non-classical antifolates and therefore their efficacy.

## Results

### 

#### 

##### RIT-seq Screens Identify Candidate Antifolate Resistance Mechanisms

**FIGURE 1.**
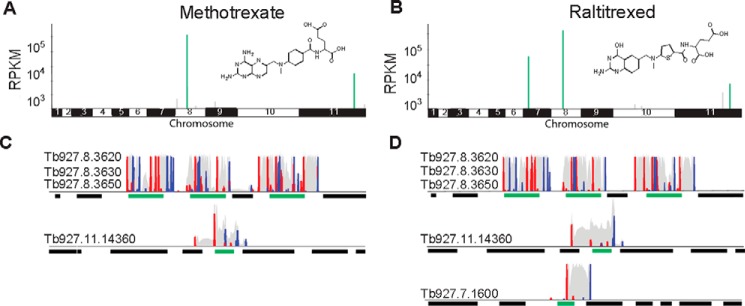
**RIT-seq screens identify candidate antifolate resistance mechanisms.** The genome-wide RIT-seq maps indicate hits from the MTX (*A*) and RTX (*B*) RNAi screens. Multiple RIT-seq fragments represent primary hits, indicated in *green*. Other loci with mapped reads are indicated in *gray*. The profiles for MTX (*C*) and RTX (*D*) indicate hits in *green*; other protein coding sequences are indicated as *black bars. Red peaks,* forward reads with RNAi-construct barcodes; *blue peaks,* reverse reads with RNAi-construct barcodes; *gray peaks,* all other reads.

A trypanosome RNAi library was exposed to a typically lethal dose of either the DHFR inhibitor, MTX, or the TS inhibitor, RTX (Fig. 1, *A* and *C*). Under tetracycline induction, each *T. brucei* cell produces dsRNA from the integrated RNAi target fragment, and knockdown has the potential to confer a selective advantage under drug pressure. RIT-seq was subsequently used to generate a readout from the population that tolerated this regime. PCR products representing RNAi target fragments, derived from the RNAi screen, were separated on an agarose gel, and low-throughput RIT-seq of multiple fragments implicated the putative folate transporter genes (*FT1–3*: Tb927.8.3620, Tb927.8.3630, and Tb927.8.3650) in both the MTX and RTX screens. These folate transporters share 96% nucleotide identity and are arranged in tandem, interrupted by an unrelated gene (Tb927.8.3640) on chromosome 8.

High-throughput RIT-seq confirmed and extended these hits as follows: 2.4 million paired-end reads, of which 70% mapped to the reference genome for MTX, and 2.6 million paired-end reads, of which 71% mapped to the reference genome for RTX (Table 1). *FT1–3* and a truncated orphan folate transporter gene (Tb11.v5.0766) are the strongest “hits” in both the MTX and RTX screens, accounting for ∼90% of all mapped reads (Fig. 1, *B* and *D*). We also note that 95% of the “MTX” and “RTX” reads mapped against a reference genome that included the *T. brucei* 427 telomeric variant surface glycoprotein expression site regions; in addition to encoding variant surface glycoproteins, these regions contain *ESAG10* genes that also encode putative folate transporters related to *FT1–3*. A gene encoding a mitochondrial carrier protein (*MCP2*, Tb927.11.14360) was also common to both screens (Fig. 1, *B* and *D,* and Table 1), whereas C-1-tetrahydrofolate synthase, also referred to as the bifunctional tetrahydrofolate dehydrogenase/cyclohydrolase (*DHCH*, Tb927.7.1600), was specific to the RTX screen (Fig. 1*D* and Table 1). A notable “hit” only supported by a single RIT-seq fragment in the RTX screen was folylpolyglutamate synthase (*FPGS*, Tb927.10.7520, Table 1) implicated in folate retention by polyglutamylation. The putative pteridine transporter genes on chromosomes 1 and 10 failed to register as hits in either screen. Thus, although pteridine transporters are members of the folate biopterin transport (FBT) family, in *T. brucei* they do not appear to be involved in the transport of antifolates.

**TABLE 1:** **Hits identified through RIT-seq screening** Hit list showing RPKM for barcoded reads (×10^3^); only genes with RPKM >1000 for barcoded reads and RPKM >200 for all mapped reads are included. MTX, methotrexate screen; RTX, raltitrexed screen.

Gene ID	Gene description	MTX reads	RTX reads
Tb927.8.3620	Folate transporter, putative	387.4*^a^*	425.6*^a^*
Tb927.8.3650	Folate transporter, putative	190.1*^a^*	139.4*^a^*
Tb927.8.3630	Folate transporter, putative	107.0*^a^*	125.5*^a^*
Tb11.v5.0766	Folate transporter, putative (truncated)	85.4*^a^*	78.9*^a^*
Tb927.11.14360	Mitochondrial carrier protein (MCP2)	16.6*^a^*	4.0*^a^*
Tb927.7.1600	C-1-tetrahydrofolate synthase, cytoplasmic, putative	0	57.3*^a^*
Tb927.11.12460	Hypothetical protein, conserved	0	3.6
Tb927.8.1040	Protein phosphatase inhibitor, putative	2.1	0.5
Tb927.9.5960	Succinate dehydrogenase, putative	1.7	0
Tb927.10.7520	Folylpolyglutamate synthase, putative (FPGS)	0	1.2
Tb927.8.5840	Hypothetical protein, conserved	1.2	0

*^a^* Primary hit with >1 fragment per gene; others, secondary hits with only one fragment per gene. GeneID and description information is from tritrypdb.org.

##### Folate and MTX Transport Kinetics

**FIGURE 2.**
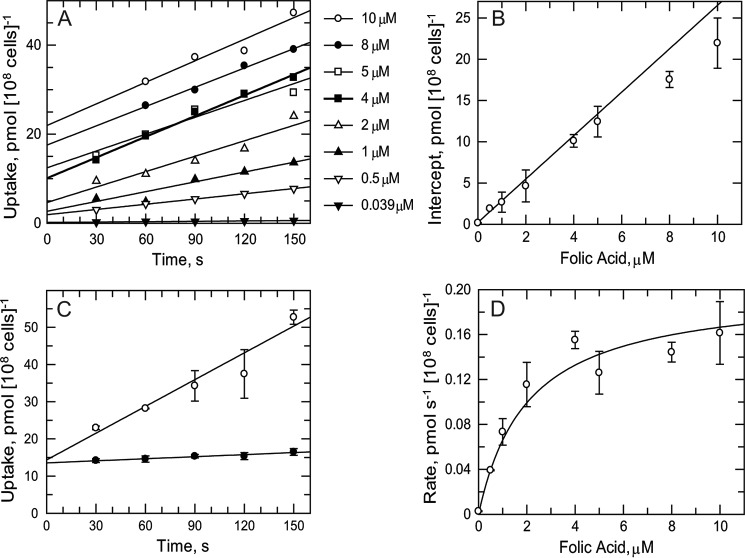
**Folate transport kinetics.** *A,* linearity of folate uptake at 23 °C. Time points at 30 s for 10 and 8 μm are not available due to loss of pellet during the experiment. *B,* intercepts (from *A*, time 0) as a function of folate concentration. Linear regression analysis uses explicit weighting for the fit to take into account the higher errors at the higher concentrations. *C,* effect of temperature on folate uptake. Trypanosomes were incubated with 10 μm folate at 23 °C (*open circles*) or 4 °C (*closed circles*) and samples processed at intervals. Data are the mean and S.E. of triplicate samples. *D,* transport kinetics for folate. Rates were obtained from the slopes of data in *A* and fitted to the Michaelis-Menten equation.

**FIGURE 3.**
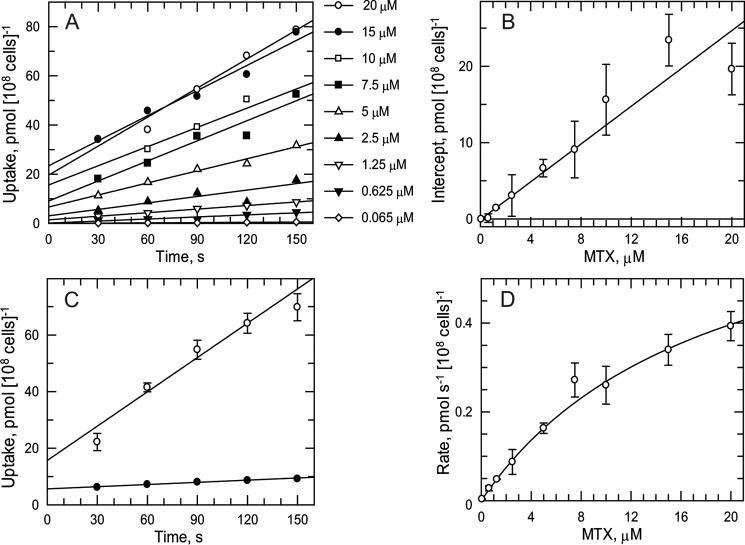
**Methotrexate transport kinetics.** *A,* linearity of MTX uptake at 23 °C. The time point at 30 s for 20 μm is not available due to loss of pellet during the experiment. *B,* intercepts (from *A*, time 0) as a function of MTX concentration. Linear regression analysis uses explicit weighting for the fit to take into account the higher errors at the higher concentrations. *C,* effect of temperature on MTX uptake. Trypanosomes were incubated with 20 μm MTX at 23 °C (*open circles*) or 4 °C (*closed circles*) and samples processed at intervals. Data are the mean and S.E. of triplicate samples. *D,* transport kinetics for MTX. Rates were obtained from the slopes of data in *A* and fitted to the Michaelis-Menten equation.

As the RIT-seq screens implicated folate transporters in antifolate drug resistance, transport studies of folate and MTX were undertaken to determine basic kinetic parameters. Folate uptake was found to be linear over 150 s and directly proportional to concentration up to 10 μm at 23 °C (Fig. 2*A*). A plot of the *y* axis intercepts (time 0) *versus* folate concentration is linear (Fig. 2*B*), suggesting nonspecific binding of folate to the trypanosome outer surface. The rate of uptake of folate was temperature-dependent (Fig. 2*C*) with a 12-fold higher rate at 23 °C (0.24 ± 0.03 pmol s^−1^ (10^8^ cells)^−1^) compared with 4 °C (0.02 ± 0.01 pmol s^−1^ (10^8^ cells)^−1^). Uptake rates for folate obeyed simple Michaelis-Menten kinetics (Fig. 2*D*) yielding a *K_m_* of 2.04 ± 0.53 μm and a *V*_max_ of 0.20 ± 0.04 pmol s^−1^ (10^8^ cells)^−1^. In the same way, MTX uptake was found to be linear over 150 s up to 20 μm (Fig. 3*A*) at 23 °C with evidence of nonspecific binding (Fig. 3*B*) and temperature dependence (Fig. 3*C* (0.40 ± 0.06 and 0.03 ± 0.01 pmol s^−1^ (10^8^ cells)^−1^) at 23 and 4 °C, respectively). MTX uptake also obeyed simple Michaelis-Menten kinetics (Fig. 3*D*) yielding a *K_m_* of 18.4 ± 2.2 μm and *V*_max_ of 0.76 ± 0.08 pmol s^−1^ (10^8^ cells)^−1^.

##### FT1–3 Transport Folate and Classical Antifolates

**FIGURE 4.**
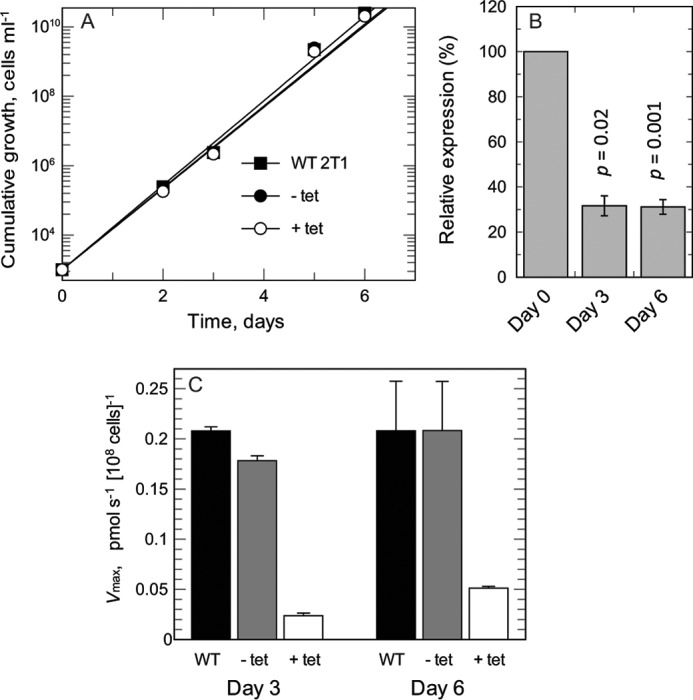
**FT1–3 knockdown and impact on folate transport.** *A,* cumulative growth of wild-type (*WT*) cells and FT RNAi strains with or without tetracycline (*tet*) induction. *Closed squares,* WT; *closed circles*, RNAi knockdown minus tetracycline induction; *open circles*, RNAi knockdown with tetracycline induction. *B,* qRT-PCR shows down-regulation of FT RNA: transcript change of FT with or without tetracycline induction, normalized against endogenous control gene *TERT. C,* folate transport kinetics measured at day 3 and day 6 in WT cells and FT RNAi strains with or without tetracycline induction.

To validate the RIT-seq analysis, we performed knockdown of FT1–3 in independent RNAi strains. Tetracycline-induced knockdown showed no significant change in growth over a 6-day period when compared with wild-type or un-induced controls (Fig. 4*A*). RNA analysis using qRT-PCR confirmed FT1–3 mRNA knockdown after 3 days of induction (*p* = 0.02) and after 6 days of induction (*p* = 0.001) (Fig. 4*B*).

RNAi knockdown of FT1–3 greatly reduced *V*_max_ of folate uptake at 3 and 6 days after induction (Fig. 4*C*). After 3 days, the *V*_max_ of induced cells was 10- and 9-fold lower than wild-type and un-induced cells, respectively. Similarly by day 6, the *V*_max_ of induced cells was 4-fold lower than wild-type and un-induced cells. No changes in *K_m_* values were detected, with *K_m_* values in the range 2.56–3.47 μm obtained for all three cell lines after 3 or 6 days of induction.

EC_50_ determinations showed a >30-fold decrease in susceptibility for MTX and RTX after 3 and 6 days of FT1–3 knockdown, respectively (Table 2). In contrast, susceptibility to the non-classical DHFR inhibitor pyrimethamine increased by 12-fold after 3 days and by 14-fold after 6 days of knockdown. Likewise, susceptibility to the non-classical TS inhibitor nolatrexed also increased by 2-fold after 3 and 6 days of knockdown. No marked change of EC_50_ for the control arsenical drug, melarsoprol, was evident after 3 or 6 days of knockdown.

**TABLE 2:** **Effect of RNAi knockdown of folate transporters FT1–3 on susceptibility to antifolates** EC_50_ values (nm) of drugs against uninduced and induced FT knockdown cells. Results are weighted means ± weighted error of three independent experiments.

Drug	3 days			6 days		
	Minus tetracycline	Plus tetracycline	-Fold shift	Minus tetracycline	Plus tetracycline	-Fold shift
Methotrexate	14.5 ± 0.4	444 ± 16.9	30.6	8.73 ± 0.39	321 ± 12.8	36.8
Raltitrexed	11.2 ± 0.3	368 ± 13.7	32.9	5.83 ± 0.20	227 ± 9.68	38.9
Pyrimethamine	2,500 ± 120	200 ± 11.0	0.08	20,40 ± 165	142 ± 10.4	0.07
Nolatrexed	36,200 ± 1,910	16,700 ± 957	0.46	39,900 ± 2,480	15,600 ± 886	0.39
Melarsoprol	1.73 ± 0.09	1.70 ± 0.08	0.98	1.26 ± 0.60	1.29 ± 0.59	1.02

##### Inhibition of Folate and MTX Transport by Alternative Substrates

**FIGURE 5.**
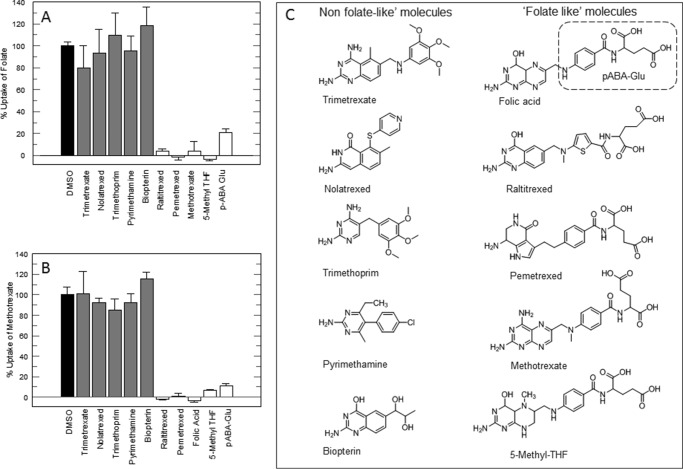
**Effect of antifolates and folate metabolites on uptake of folate or MTX.** Uptake of folate (*A*) and MTX (*B*) was measured in the presence of 100 μm of each inhibitor. *Black bar,* DMSO control; *gray bars,* non-folate-like structures; *white bars,* folate-like structures. The standard assay mixture is modified to contain [S] ≅ *K_m_* and corrected for additional DMSO (0.4%). *C,* chemical structures of drugs and metabolites.

To further assess the interactions between trypanosomes and antifolates, we determined whether “folate-like molecules” had an effect on folate or MTX uptake. Folate uptake was inhibited by MTX, RTX, pemetrexed, and 5-methyl-THF (Fig. 5*A*). In contrast, uptake of folate was not inhibited by compounds lacking a pABA-Glu-like moiety, namely trimetrexate, nolatrexed, trimethoprim, pyrimethamine, or biopterin (see Fig. 5*C* for chemical structures). Consistent with this observation, pABA-Glu itself substantially reduced folate uptake (21% residual uptake). MTX uptake was inhibited by the same classical antifolates or folic acid but not by the non-classical antifolates (Fig. 5*B*). As for folate, pABA-Glu also substantially reduced MTX uptake (11% residual uptake). Biopterin did not inhibit folate or MTX uptake, in agreement with the failure of the RIT-seq screens to identify the putative pteridine transporter genes on chromosomes 1 and 10.

**FIGURE 6.**
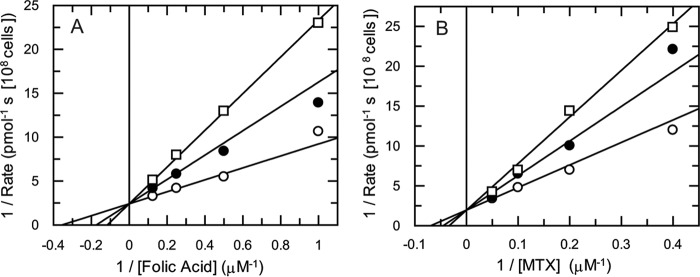
**Mode of inhibition of folate transport by MTX and vice versa.** *A, K_i_* determination of MTX with respect to folate. Folate concentrations were varied in the presence of fixed concentrations of MTX. MTX was added at 0 μm (*open circles*), 0.5 μm (*closed circles*), and 1 μm (*open squares*). *B, K_i_* determination of folate with respect to MTX. Folate was added at 0 μm (*open circles*), 0.5 μm (*closed circles*), and 1 μm (*open squares*), and MTX was the variable substrate. The lines are best fits to linear competitive inhibition (Equation 1).

Finally, we determined the IC_50_ values of folate analogues against uptake of either radiolabeled folate or MTX. MTX inhibited folate uptake with an IC_50_ of 3.4 ± 0.4 μm, and folic acid inhibited uptake of MTX with an IC_50_ of 1.9 ± 0.4 μm (Table 3). The classical antifolates RTX and pemetrexed had similar potencies to MTX in inhibiting folate uptake. pABA-Glu was a weaker inhibitor of both folate and MTX uptake (IC_50_ values of 30.8 ± 3.7 and 33.5 ± 4.6 μm, respectively) (Table 3). To determine whether MTX competes with folate for uptake, transport rates were determined for varying concentrations of folate in the presence of fixed concentrations of MTX (Fig. 6*A*). The resulting Lineweaver-Burk plot suggested either a competitive or mixed inhibition pattern. An *F*-test in GraFit established that these data fitted best to competitive inhibition, yielding a *K_i_* value for MTX of 0.48 ± 0.07 μm. A similar analysis revealed that folate is a competitive inhibitor of MTX transport with a *K_i_* value of 0.94 ± 0.21 μm (Fig. 6*B*). If two substrates compete for the same target or enzyme, in this case the folate transporter, Equations 6 or 7 (see derivation under “Experimental Procedures”) can be used to calculate the IC_50_ for any concentration of ligand. Applying experimental values to these equations yielded an IC_50_ for MTX of 2.2 μm against folate uptake (Equation 6) and an IC_50_ value for folate of 2.9 μm against methotrexate uptake (Equation 7). These values are in good agreement with the EC_50_ values determined experimentally (Table 3), thus giving further evidence of a competitive substrate model for folate and MTX transport by FT1–3.

**TABLE 3:** **Uptake of folate and MTX in the presence of varying concentrations of inhibitor** The standard assay mixture was modified to contain [S] ≅ *K_m_* and corrected for additional DMSO (0.4%). IC_50_ was calculated by fitting to a two-parameter IC_50_ equation. ND = not determined.

Substrate	IC_50_ against folate	IC_50_ against MTX	*K_m_*	*V*_max_	Calculated IC_50_
	μ*m*	μ*m*	μ*m*	*pmol s*^−*1*^ *(10^8^ cells)*^−*1*^	μ*m*
Methotrexate	3.4 ± 0.4		16.5	0.68	2.2
Folic acid		1.9 ± 0.4	2.07	0.19	2.9
5-Methyl-THF	6.3 ± 1.0	8.0 ± 0.8			
pABA-Glu	30.8 ± 3.7	33.5 ± 4.6			
Pemetrexed	3.2 ± 0.3	ND			
Raltitrexed	1.8 ± 0.4	ND			

## Discussion

Our experiments have identified functional folate transporters in *T. brucei*. The folate biopterin transporter (FBT) family is a novel class of membrane proteins and superfamily of transporters that were first characterized in *Leishmania* (21, 22) and are found in other kinetoplastids (23) (including *T. brucei*), malaria (24), *Toxoplasma* (25), plants (26) and cyanobacteria (26). The *T. brucei* reference genome encodes eight FBT genes as follows: in tritrypdb.org (version 9.0), four of are annotated as putative folate transporters, with FT1–3 (Tb927.8.3620, Tb927.8.3630, and Tb927.8.3650), clustered together on chromosome 8 and a truncated orphan gene on chromosome 11 (Tb11.v5.0766); and four annotated as putative pteridine transporters, with three (Tb927.1.2820, Tb927.1.2850, and Tb927.1.2880) clustered together on chromosome 1 and an orphan gene on chromosome 10 (Tb927.10.9080). The putative folate transporters are predicted by the TMHMM server version 2.0 to contain 12 transmembrane helices consistent with other folate transporters. The orphan FT gene (Tb11.v5.0766) shares 97–100% sequence identity to *FT1–3* (position 736–1899 of ORF), but this gene encodes a protein that lacks the first 245 amino acid residues that are predicted to encode the first four transmembrane segments and therefore is likely to be non-functional. There are also seven expression site-associated genes (*ESAG10*) in the *T. brucei* S427 strain that share 84–91% amino acid identity with FT1–3. *ESAG10s* are located in about half of the specialized telomeric transcription units (Bloodstream Expression Sites (BES)) devoted to expression of variant surface glycoproteins (27). Only one BES is actively transcribed at any time, and our lines express VSG221 from a BES that lacks *ESAG10* (27). VSG221 expression is checked periodically and is consistently found to be the case for >99% of these cells. Thus, FT1–3 are likely the only expressed and functional folate transporters in bloodstream-form *T. brucei*.

In contrast to the seven FBT homologues in *T. brucei*, *Leishmania* has 14 FBTs, and the function of 3 members, BT1, FT1, and FT5 is known. FT5 is a high affinity/low capacity transporter of folate/MTX (21), and FT1 is the main folate transporter in *Leishmania* (high affinity/high capacity for folate/MTX) (28). In contrast, BT1 is a biopterin transporter that mediates uptake of biopterin and folate but not MTX (29). From our experiments in *T. brucei,* folate and MTX share the same transporter system, FT1–3, which is not shared by biopterin, a similar finding to that in *Leishmania*. It would be interesting to establish whether the other four members of the *T. brucei* FBT family do indeed mediate uptake of biopterin. In *Leishmania*, the FBT family was annotated in tritrypdb.org as pteridine transporters; however, there is a fourth member of this gene family, AdoMetT1, that has recently been discovered to mediate the specific transport of *S*-adenosylmethionine (30).

RNAi knockdown of FT1–3 dramatically reduced folate transport, and competition assays with antifolates indicated that folate and classical antifolates share a common transport mechanism via folate transporters, which is not the case for non-classical antifolates. Indeed, classical antifolates bear a close structural resemblance to folate and use the same mechanism as folates for cell entry via specific transporters in human cells; in contrast, non-classical antifolates are lipophilic, do not contain a terminal glutamyl moiety, and enter cells by passive or facilitated diffusion (31–33). In our experiments involving knockdown of FT1–3, uptake of both folate and classical antifolates was reduced, leading to depletion in intracellular drug levels and loss of potency against the intracellular drug target DHFR-TS (13). However, in the case of non-classical antifolates, uptake of the drug is not affected by knockdown, whereas uptake of folate is reduced, leading to diminished intracellular folate to compete for the drugs' target (DHFR-TS) and therefore improved drug potency.

Our competition assay also provided further insight into the structural recognition of folate for its uptake via transporters. pABA-Glu inhibited folate and MTX transport, whereas biopterin had no effect on transport. This indicates that structures that contain pABA-Glu (5-methyl-THF, folate) or a pABA-Glu-like moiety (classical antifolates) compete for uptake into cells (Fig. 5*C*). Indeed the α- and γ-glutamate carboxyl groups of the classical antifolates are negatively charged at physiological pH and thus require carrier-mediated uptake for entry into cells (34). The addition of a pABA-Glu moiety to a non-classical antifolate, such as pyrimethamine, could lead to improved drug uptake via transport and might also compete for folate uptake, potentially leading to increased drug potency. Conversely, one advantage of non-classical lipophilic antifolates over classical antifolates is the reduced risk of transport-related resistance occurring (35, 36). This observation has implications for future chemotherapy. Drug resistance in *T. brucei* can arise due to deletions disrupting genes encoding drug transporters as follows: *AQP2* and *AT1* in the case of melarsoprol resistance (37, 38) and *AAT6* in the case of eflornithine resistance (12). A similar mechanism may lead to antifolate resistance, in which case a strategy of rotating between classical and non-classical antifolates or using a combination of classical and non-classical antifolates for chemotherapy may minimize resistance emerging. Alternative resistance mechanisms involving overexpression of target enzymes in *Leishmania* (39, 40) or mutation in target enzymes in malaria parasites (41) have been described, however.

The properties of folate transport in the *T. brucei* bloodstream form are quite similar to that found in *Leishmania,* where uptake is rapid and linear for 2–3 min in both parasites (42) The rate of uptake is also concentration-dependent, highlighting a carrier-mediated mechanism of transport. Both transport systems exhibit Michaelis-Menten kinetics, with *K_m_* value for *Leishmania* parasites in the high nanomolar to low micromolar range, depending upon the species (42). In contrast, mammalian cells show a much lower affinity for uptake of folic acid (100-fold less affinity that in *T. brucei*) and have a very high affinity for 5-methyl-THF, the principal form of folate in human serum (43). The *K_m_* or *K_i_* values for 5-methyl-THF transport in mammalian tumor cells (range 1–4 μm) (44) is comparable with the *K_m_* of folate in *T. brucei* (2.1 μm). Likewise, *T. brucei* was shown to have a lower affinity for MTX uptake with a *K_m_* of 16 μm, as is the case for mammalian cells (range 2.3 to 26 μm) (44).

As well as shedding light on the role of FT1–3 in MTX and RTX drug resistance, which led to the exploration of transport kinetics of folate and antifolate drugs, our RIT-seq experiments also identified other hits of interest. The mitochondrial carrier protein 2 (MCP2) was a hit in both drug screens Mitochondrial carrier proteins are a group of structurally conserved proteins that regulate the transport of a variety of metabolic intermediates across the mitochondrial membrane; 24 mitochondrial carrier proteins have been identified in *T. brucei* (45). Phylogenetic reconstruction shows TbMCP2 on a branch with the human folate transporter, SLC25A32, and TbMCP2 has been confirmed as mitochondrial by Myc tagging (45). One-carbon metabolism in mammalian cells is compartmentalized in the cytosol, mitochondrion, and nucleus (46). However, the subcellular localizations of key enzymes in folate-dependent pathways have yet to be fully elucidated in African trypanosomes. Methionyl-tRNA^Met^ formyltransferase, required for initiation of mitochondrial protein biosynthesis, has been localized experimentally to this organelle (47). In addition, proteomic analysis of enriched mitochondrial preparations from *T. brucei* identified all components of the glycine cleavage system as mitochondrial (48). Other folate enzymes (DHFR-TS and DHCH) were identified by enrichment, but a mitochondrial location has not been confirmed. Underlining this uncertainty, DHCH is localized exclusively in the cytosol in the related trypanosomatid, *Leishmania major* (49). Nonetheless, the fact that at least some folate-dependent metabolic enzymes have a confirmed mitochondrial location indicates that folates must be transported into this organelle.

Notably, DHCH and the “minor hit” FPGS were specific to RTX in RIT-seq experiments. DHCH is a bifunctional enzyme catalyzing formation of THF from *N*^5^,*N*^10^-methylene-THF in a two-step reaction (50). Decreased DHCH activity would result in accumulation of *N*^5^,*N*^10^-methylene-THF and increased conversion of dUMP to dTMP by TS for DNA synthesis, thus providing an additional mechanism of resistance to RTX, a TS-targeted antifolate. Moreover, RTX has a terminal glutamyl moiety that is polyglutamylated *in vivo* by FPGS (51). RTX is fully active after polyglutamylation (52) resulting in tighter binding to its target TS and improved cellular retention. RTX has shown to be more potent against whole parasites than against recombinant TbDHFR or TbTS (13) suggesting that polyglutamylation is likely to occur inside *T. brucei*. Thus, we propose that this is the mechanism by which FPGS knockdown reduces RTX potency.

An alternative approach to drug discovery is therapeutic switching, *i.e.* the utilization of known approved drugs already used in the treatment of many other human diseases. Indeed, there are many antifolate drugs that are clinically in use, mainly developed as anticancer, antimalarial, and antibacterial drugs that have also shown success in the treatment of toxoplasmosis as well as promising potential against cryptosporidiosis (53). Most are available as oral preparations and many have good central nervous system penetration; these two pharmacological properties concur with the goal of Drugs for Neglected Diseases initiative of delivering a new oral only treatment for stage 2 sleeping sickness to the clinic (54). The folate biosynthetic pathway in *T. brucei* has yet to be fully exploited, and future endeavors to understand the underlying mechanisms involved in these pathways will play a central role when designing and refining new and existing antifolates. Our studies have identified a key role of the folate transporter genes, FT1–3, in the uptake of folate and folate analogues. This provides new insight into potential resistance mechanisms and chemotherapy strategies using antifolates.

## Experimental Procedures

### 

#### 

##### Chemicals

Folic acid, biopterin, and *p*-amino benzoyl-l-glutamic acid were purchased from Schircks Laboratories. [^3^H]Folic acid (64 Ci mmol^−1^) and [^3^H]MTX (38.2 Ci mmol^−1^) were purchased from Hartmann Analytical. DHFR-TS inhibitors were sourced as follows: MTX, trimethoprim and pyrimethamine from Sigma; nolatrexed, pemetrexed, and RTX from Sequoia Research Products; and trimetrexate from Tocris Bioscience. Melarsoprol was obtained from Rhone-Poulenc. Other chemicals and reagents used in this study were of the highest grade and purity available.

##### Trypanosomes and Culture Media

For routine culture, *T. brucei* bloodstream-form “single marker” S427 (55) and 2T1 (56) strains were cultured at 37 °C in the presence of 5% CO_2_ in HMI9-T medium. HMI9-T medium contains high concentrations of thymidine (∼160 μm) and folate (∼9 μm), the latter principally from Iscove's modified Dulbecco's medium and 10% Serum Plus components (14). A medium based on HMI9-T, lacking Serum Plus, folate, and thymidine, and using 200 μm 2-mercaptoethanol in place of 56 μm 1-thioglycerol, was prepared in-house and named *T. brucei* base media (TBM) (13). Residual folate in TBM is provided by the 10% fetal calf serum component. Wild-type S427 and 2T1 *T. brucei* cells grow normally in TBM, and the rate of growth is similar to HMI9-T (7–8-h doubling time).

##### RNAi Screening and RIT-seq

An RNAi library screen was performed in TBM and carried out as described previously (57). Briefly, the RNAi library was induced on day 0 with tetracycline (1 μg ml^−1^) and maintained under blasticidin (1 μg ml^−1^) and phleomycin (1 μg ml^−1^) selection at a minimum of 2.5 × 10^7^ cells in 150 ml of media. Following induction for 24 h, 15 nm MTX or 10 nm RTX (∼3–4× EC_50_ for parasites cultured in TBM for 48 h) was added. Cultures were split and supplemented with fresh drug as required. At day 4, concentrations of MTX and RTX were increased to 30 nm. DNA was extracted from drug-resistant cells on day 10. RNAi target fragments were then amplified by PCR using the LIB2f and LIB2r primers. For low throughput identification of fragments, PCR products were separated on an agarose gel, and individual bands were excised and sequenced using the Lib2f and Lib2r primers (57). For high throughput identification of fragments, the PCR products were fragmented and sequenced using an Illumina HiSeq platform at BGI (Beijing Genomics Institute). Reads were mapped to the *T. brucei* 927 reference genome (v9.0, tritrypdb.org) with Bowtie 2 (58) using the following parameter: very-sensitive-local-phred33. The generated alignment files were manipulated with SAMtools (59) and a custom script to identify reads with barcodes (GCCTCGCGA) (57). Total and bar-coded reads were then quantified using the Artemis genome browser (60). Hit-lists generated from RIT-seq analyses excluded selected large gene families, including variant surface glycoproteins, and genes immediately adjacent to hits.

##### T. brucei RNAi Constructs and Strains

PCR primers were designed using RNAit (61) to generate a 536-bp fragment conferring specific knockdown to FT1–3 (forward, GATCGGGCCCGGTACCGCTTGTGAGTTGGGTTTGGT, and reverse, GATCTCTAGAGGATCCCGATCACAAGTGGAAGAGC). PCR fragments were cloned in the pRPa^SLi^ construct for the generation of stem-loop dsRNA under the control of tetracycline as the trigger for RNAi (56, 62). Constructs were digested with AscI, ethanol-precipitated, and resuspended (1 μg ml^−1^) in sterile water. 2T1 strains, containing a tetracycline repressor (56), were electroporated using program X-001 of the Nucleofector II electroporator (Amaxa, Cologne, Germany) (63) following the addition of 5 μg of linearized DNA mixed in 100 μl of cytomix (64). Transformants were cloned by limiting dilution under phleomycin (1 μg ml^−1^) and hygromycin (2.5 μg ml^−1^) selection. Puromycin susceptibility (1 μg ml^−1^) was tested for full integration of the construct, and expression of stem-loop RNAi was induced with 1 μg ml^−1^ tetracycline.

##### Quantitative RT-PCR

*T. brucei* RNA was isolated using an RNeasy purification kit (Qiagen) and cDNA-synthesized using a high capacity RNA-to-cDNA kit (Applied Biosystems). PCR primers were designed using the Premierbiosoft's Beacon Designer 6 to amplify a 117-bp region common to all three FT genes but distinct from the region targeted by RNAi (forward, GAATTGCTGACAACATCATT, and reverse, TCACTGCGTAACCAAATGTA). qRT-PCRs consisted of 1 μl (40 ng) of cDNA, 10 μl of Brilliant III Ultra-Fast QPCR Master Mix (Agilent Technologies), 1 μl (500 nm) each of the forward and reverse primers, and 0.3 μl (30 nm) of reference dye and nuclease-free PCR grade-treated water. PCR was performed using an Agilent Mx3005P machine with the following cycling conditions: 95 °C for 3 min; 40 cycles of 95 °C for 20 s; then 60 °C for 20 s. The reference gene *TERT* (Tb927.11.10190, telomerase reverse transcriptase) was used to provide a baseline of transcription levels for normalization of the data (forward, AGGAACTGTCACGGAGTTTGC, and reverse, AGGAACTGTCACGGAGTTTGC). Relative quantification in the tetracycline-induced FT1–3 knockdown cell line was normalized to the un-induced cell line using the ΔΔ*Ct* method, and a Student's unpaired *t* test was used to show significance on four experimental replicates. Statistical analyses were performed using Excel and GraFit 5.013 (Erithacus software).

##### EC_50_ Determination of Antifolates

EC_50_ of antifolates in FT knockdown lines were determined after 3 and 6 days of induction with tetracycline in TBM. Serial doubling dilutions of antifolates (5–50 mm stocks prepared in DMSO) were prepared in 96-well microtiter plates in 100 μl of TBM, and trypanosomes (resuspended in the same medium) were added in 100 μl to give a final density of 2.5 × 10^3^ cells ml^−1^ in 96-well plates. All wells, including controls, contained a final volume of 0.5% DMSO. Cultures were incubated for 72 h at 37 °C, 5% CO_2_ before cell density was determined using a resazurin-based assay (65). EC_50_ values were calculated using GraFit version 5.0.13 (Erithacus Software) with a 3-parameter fit from triplicate readings. A weighted mean from three independent experiments was calculated.

##### Transport Assay

*T. brucei* cells were grown in TBM for 72 h at an initial seeding density of 5 × 10^3^ cells ml^−1^. Cells were harvested by centrifugation (800 × *g*, 10 min, 4 °C), washed, and resuspended in transport buffer (33 mm HEPES, 98 mm NaCl, 4.6 mm KCl, 0.55 mm CaCl_2_, 0.07 mm MgSO_4_, 5.8 mm NaH_2_PO_4_, 0.3 mm MgCl_2_, 23 mm NaHCO_3_, 14 mm glucose, pH 7.3) (66) at a density of 2.5 × 10^8^ cells ml^−1^. Transport assays were carried out as described previously by Ong *et al.* (67). Uptake was initiated by mixing 100 μl of cells with 100 μl of transport buffer containing 0.5 μCi of radiolabeled ligand (and potential inhibitors of transport, where indicated) and layered over 100 μl of dibutyl phthalate (Sigma) in a 1.5-ml microcentrifuge tube. Transport was stopped by centrifugation of cells through the dibutyl phthalate layer (16,000 × *g* for 1 min). Microcentrifuge tubes were flash-frozen in liquid nitrogen, and the bottom of the tubes containing the cell pellets were cut off directly into scintillation vials. Pellets were solubilized in 1 m NaOH (150 μl) overnight and mixed with scintillation fluid (2 ml), and radioactivity was measured using a liquid scintillation counter (Beckman Coulter).

##### Transport Kinetics

Uptake of [^3^H]folic acid (0.04 μm) was determined in the presence of varying concentrations of unlabeled folate. Similarly, uptake of [^3^H]MTX (0.07 μm) was determined in the presence of varying concentrations of unlabeled MTX. Uptake of both folate and MTX was determined at regular time intervals (30, 60, 90, 120, and 150 s) at 23 °C and fitted using robust non-linear fitting to the linear equation *y* = *mx* + *c*. The nonspecific binding of radiolabeled ligand to trypanosomes at 4 °C was determined in a similar fashion. To calculate *K_m_*, results were fitted by non-linear regression to the Michaelis-Menten equation. A weighted mean from three independent experiments was calculated.

##### Folate and MTX Inhibition Studies

Linear rates of uptake were measured as before in the presence of 100 μm competing inhibitor with the standard assay mixture modified to contain folate (2.0 μm) or MTX (13 μm) at approximately [S] = *K_m_*. Inhibitor concentrations giving 50% inhibition (IC_50_) were then determined over a range of concentrations across 7–8-point serial doubling dilutions. Dose-response curves were fitted by non-linear regression to a two-parameter IC_50_ equation using GraFit 5.0 (Erithacus software). To determine the mode of inhibition of MTX on folate uptake and folate on MTX uptake, the linear rate of uptake was measured as before using four different substrate concentrations and three different inhibitor concentrations. The resulting data were plotted as a Lineweaver-Burk transformation, and the graphs were inspected to establish the most likely mode of inhibition (intersection on *y* axis). An *F* test confirmed the mode of inhibition as competitive rather than mixed. The entire data set was then globally fitted to the competitive Equation 1.

(Eq. 1) $$v\;=\;\frac{V_{\max}\left[\text{S}\right]}{K_{m}\left(1+\frac{\left[1\right]}{K_{i}}\right)\;+\;\left[\text{S}\right]}$$

If folate and methotrexate are competitive substrates for the same transporter, then, for folate we obtain Equation 2,

(Eq. 2) $$v_{\text{F}}\;=\;\frac{V_{\max}^{\text{F}}\left[\text{F}\right]}{K_{m}^{\text{F}}\left(1+\frac{\left[\text{M}\right]}{K_{m}^{\text{M}}}\right)\;+\;\left[\text{F}\right]}=\frac{\left(V_{\max}^{\text{F}}/K_{\text{m}}^{\text{F}}\right)\left[\text{F}\right]}{1\;+\;\frac{\left[\text{M}\right]}{K_{m}^{\text{M}}}\;+\;\frac{\left[\text{F}\right]}{K_{m}^{\text{F}}}}$$

and for methotrexate we get Equation 3,

(Eq. 3) $$v_{\text{M}}\;=\;\frac{V_{\max}^{\text{M}}\left[\text{M}\right]}{K_{m}^{\text{M}}\left(1+\frac{\left[\text{F}\right]}{K_{m}^{\text{F}}}\right)\;+\;\left[\text{M}\right]}=\frac{\left(V_{\max}^{\text{M}}/K_{\text{m}}^{\text{M}}\right)\left[\text{M}\right]}{1\;+\;\frac{\left[\text{F}\right]}{K_{m}^{\text{F}}}\;+\;\frac{\left[\text{M}\right]}{K_{m}^{\text{M}}}}$$

where [M] and [F] refer to the concentrations of methotrexate and folate, respectively. Dividing Equation 3 by Equation 2 yields Equation 4,

(Eq. 4) $$\left(\frac{v_{\text{M}}}{v_{\text{F}}}\right)\;=\;\left(\frac{\left(V_{\max}^{\text{M}}/K_{m}^{\text{M}}\right)\left[\text{M}\right]}{\left(V_{\max}^{\text{F}}/K_{\text{m}}^{\text{F}}\right)\left[\text{F}\right]}\right)$$

The IC_50_ for methotrexate ([M] = IC_50_^M^) is defined in Equation 5 when

(Eq. 5) $$\left(\frac{v_{\text{M}}}{v_{\text{F}}}\right)\;=0.5$$

Substituting Equation 5 into Equation 4 and rearranging yields Equation 6

(Eq. 6) $${\text{IC}}_{50}^{\text{M}}\;=\;\frac{0.5\left(V_{\max}^{\text{F}}/K_{\text{m}}^{\text{F}}\right)\left[\text{F}\right]}{\left(V_{\max}^{\text{M}}/K_{\text{m}}^{\text{M}}\right)}$$

Likewise, the IC_50_ for folate, defined in Equation 7, can be obtained from Equation 4 when *v*_F_/*v*_M_ = 0.5,

(Eq. 7) $${\text{IC}}_{50}^{\text{F}}\;=\;\frac{0.5\left(V_{\max}^{\text{M}}/K_{\text{m}}^{\text{M}}\right)\left[\text{M}\right]}{\left(V_{\max}^{\text{F}}/K_{\text{m}}^{\text{F}}\right)}$$

## Author Contributions

S. D. conducted the experiments, analyzed the results, and wrote the first draft of the paper. N. S. and H. B. O. designed the folate-deficient medium and assisted in the design of the transport experiments. R. J. W. and D. H. designed the RIT-seq experiments and helped analyze the data. A. H. F. conceived the project and analyzed all data. All authors contributed to writing the paper.

## Footnotes

3 The abbreviations used are:

HAT: human African trypanosomiasis
DHFR-TS: dihydrofolate reductase- dihydrofolate reductase
PTR1: pteridine reductase 1
FPGS: folylpolygluamate synthase
MTX: methotrexate
RTX: raltitrexed
RIT-seq: RNA interference target sequencing
pABA-Glu: *p*-aminobenzoylglutamate
DHCH: tetrahydrofolate dehydrogenase/cyclohydrolase
THF: tetrahydrofolate
MCP: mitochondrial carrier protein
FBT: folate biopterin transport
BES: Bloodstream Expression Site
qRT: quantitative RT
TBM: *T. brucei* base medium
TS: dihydrofolate reductase
FT: folate transporter.
